# Implications of intra-plot heterogeneity for yield estimation accuracy: Evidence from smallholder maize systems in Ethiopia

**DOI:** 10.1016/j.fcr.2021.108147

**Published:** 2021-06-15

**Authors:** Tesfaye Shiferaw Sida, Jordan Chamberlin, Hailemariam Ayalew, Frederic Kosmowski, Peter Craufurd

**Affiliations:** a International Maize and Wheat Improvement Center (CIMMYT), ILRI, P.O. Box 5689, Addis Ababa, Ethiopia; b International Maize and Wheat Improvement Center (CIMMYT), Nairobi, Kenya; c Trinity College Dublin (TCD), Dublin, Ireland; d CGIAR Standing Panel on Impact Assessment (SPIA), Hanoi, Viet Nam; e International Maize and Wheat Improvement Centre, Kathmandu, Nepal

**Keywords:** Yield variability, Within plot variation, Agronomic decision, Farm intensification, Inter-plot heterogeneity

## Abstract

Intra-plot heterogeneity in yield is often observed in smallholder farming systems, although its implications for yield measurement remain under-investigated. Using a unique dataset on smallholder maize production in Ethiopia, we quantify the magnitude of inter- and intra-plot heterogeneity, describe the relationship between intra-plot heterogeneity and maize productivity, and document the implications of intra-field heterogeneity on the accuracy of alternative yield estimation protocols. Our data include five common yield estimation protocols, as well as full plot harvests of 230 smallholder maize fields. We surveyed agronomic decisions, biophysical variables, and accessibility characteristics of the surveyed fields. We quantify intra-plot heterogeneity using the coefficient of variation (CV) of stand density, cob weight, and maize grain yield. A generalized linear mixed model is used to explore the relationship between these variables and the method- and heterogeneity-dependence of yield estimation accuracy. We find inter-plot CV values ranging from 32 to 56 %, 22 to 73 % and 39 to 49 % in population density, cob weight and grain yield, respectively. Intra-plot heterogeneity constituted most of this variation, with across-method mean CV values of 41 %, 82 % and 63 %, respectively, of the total variability in population density, cob weight and grain yield. A rise in intra-plot heterogeneity of 0.5 % to 0.8 % is associated with a significant increase in yield estimation error under alternative yield estimation protocols. Regression analysis shows that interactions in agronomic decisions, input intensity and plot accessibility factors dictate intra-plot heterogeneity and method accuracy in smallholder systems. Intra-plot heterogeneity is larger than inter-plot heterogeneity in the current study area. Our analysis shows that the effect of intra-plot heterogeneity on yield estimation accuracies is method-dependent and yield estimation methods that fail to capture true intra-plot heterogeneity are more error-prone. Results of such estimations should be considered with caution when used as the basis of decision-making.

## Highlights

• We examine inter- and intra-plot heterogeneity of productivity in maize plots.
• We find high inter- and intra-plot variabilities in plant population and yield.
• Intra-plot heterogeneity overrides inter-plot heterogeneity in smallholder maize.
• The magnitude and distribution of yield estimation errors are method-dependent.
• More intensively managed plots show lower intra-plot heterogeneity and lower yield.

## 1 Introduction

Smallholder agriculture sustains close to 60 % of the global rural population (Zavatta, 2014) and 70 % of the rural population in sub-Saharan Africa (SSA) as a primary source of livelihood (Davis et al., 2017). It contributes about 50 % of the national GDP in SSA (Kour and Arora, 2020) and is posited to be the direct driver for a number of Sustainable Development Goals (SDGs) in the continent (Sachs et al., 2019). Improving the productivity of these systems has been a central agenda for governments, donors and research organizations. The goal of improving smallholder productivity represents a primary component in the effort to reduce poverty, achieve food security and meet many SDGs. Accurate yield measurements enable the monitoring of trends in agricultural production by governments and international institutions (Nsiah and Fayissa, 2019), the planning and execution of interventions in agricultural development by research and donor organizations (Farrington et al., 1993), the development of timely early warning systems for disaster and risk management (Basso et al., 2013), and the design of optimum agronomic recommendations (Djurfeldt et al., 2018). Failure to acquire correct agricultural information leads to misinformed policy formulations, resulting in inefficient allocation of scarce resources, hence an inability to tackle acute development challenges in many countries of SSA (Kelly et al., 1995).

Attempts to estimate crop yield have been around for more than half a century, starting from the 1940s (FAO, 1982), and inquiries into the accuracy of yield estimation methods in smallholder systems remain active (Burke and Lobell, 2017; Chivasa et al., 2017; Ngoune Tandzi and Mutengwa, 2020; Tewes et al., 2020). Between- and within-field heterogeneity has been recognized as a vast challenge in making accurate yield estimations in smallholder farming systems of SSA (Bevis and Barrett, 2020; Fermont and Benson, 2011; Joshi et al., 2019; Wahab, 2019), although the exact effect of intra-plot heterogeneity on yield estimation accuracy has been less quantified. Inter-plot heterogeneity is reported to explain up to 60 % variation in maize yields of smallholder farms (Tittonell et al., 2007). Such heterogeneity can be prompted by natural and management-induced variability in soil fertility (Tittonell et al., 2013), selective input intensity (Tittonell et al., 2007), between field variation in agronomic management (Goshu et al., 2015) and other factors affecting stand establishment (Sussy et al., 2019). Socio-economic factors such as labor availability for weeding and access to intensive inputs such as fertilizer, improved seed and irrigation facilities can affect the magnitude of intra-plot heterogeneity in smallholder maize systems. According to Singh et al. (2013) even fields with a long history of consistent management can show significant within-field soil variability, as evidenced by a 30 % coefficient of variation, for soil fertility parameters sampled from single fields. Interestingly, within-field heterogeneity in soil, which is expected to drive heterogeneity in yield outcomes, is often acknowledged (Joshi et al., 2019; Tittonell et al., 2007), but rarely quantified in smallholder maize systems of SSA. Efforts such as Wahab (2019), which clearly identified in-season shrinkage of crop area as a driver of the intra-plot variability in crop performance are uncommon. Studies identifying other drivers of intra-plot variability and its effect on yield estimation accuracy are even scarcer.

The existence of intra-plot heterogeneity could have a direct bearing on input allocation, differential agronomic management and the productivity of these systems. Indirectly, it may influence policy formulation as it, for example, relates to reforms targeting land-use planning, food security and identification of agroecologies for agricultural development programs, such as the Agricultural Growth Program (AGP) in Ethiopia (Bachewe et al., 2018). Nonetheless, how this heterogeneity affects yield assessment remains poorly understood. Although it has been established that sample-based yield estimates, including common methods like crop cuts, are not error-free (Zheng et al., 2003), how these errors vary with the magnitude of intra-plot heterogeneity is unclear. Becides, the magnitude, distribution and correlates of the intra-plot heterogeneity need better description, as do the implications of intra-plot heterogeneity for the measurement of yields. If different yield estimation protocols perform differently under different degrees of heterogeneity, then documentation of such differences may help to inform the optimal design of yield estimation efforts in maize systems with differing production characteristics. This paper aims to answer four related questions:

1) What is the magnitude and distribution of within-field (intra-plot) heterogeneity in smallholder maize systems?
2) What are the main agronomic, input, biophysical and plot accessibility correlates/drivers of intra-plot heterogeneity in smallholder maize systems?
3) Which yield estimation methods perform best under inherently heterogeneous smallholder maize farming systems?
4) What is the relationship between intra-plot heterogeneity and land productivity measures?

## 2 Methodology

### 2.1 Site description and experimental setting

**Fig. 1:**
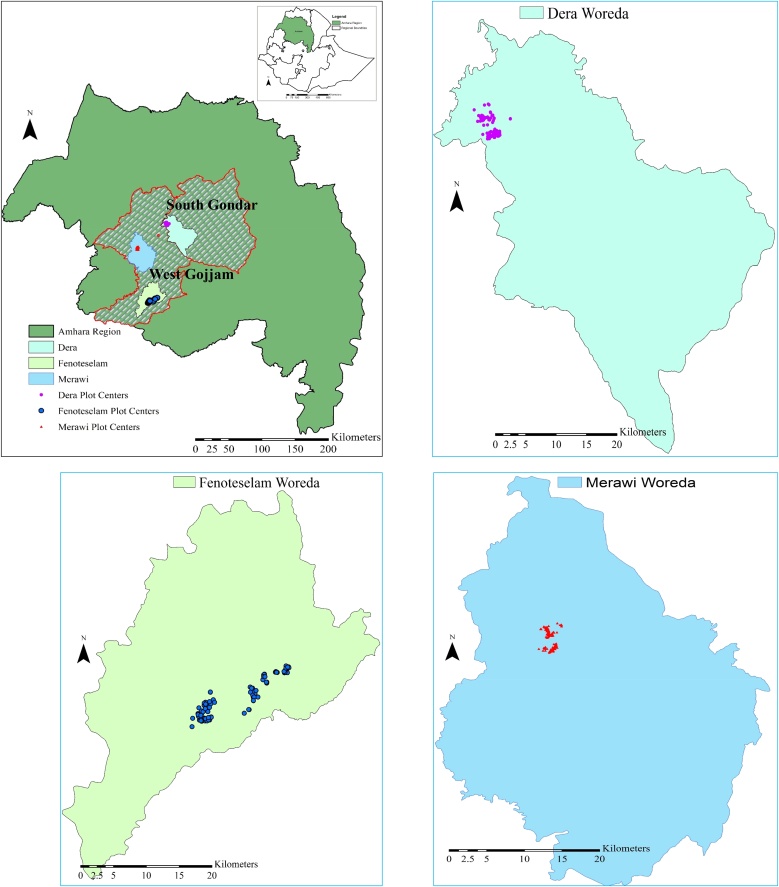
Map of study area showing the Amhara region, the two zones, the three Woreda's and the points indicating the centroid of study plots.

We selected three woredas (Dera, Fenoteselam and Merawi) in two zones (South Gondar and West Gojjam) of Ethiopia’s Amhara region in the northwestern maize belt of the country (Fig. 1). Within these areas, we consulted local extension agents and selected farmers who had ready-to-harvest plots for participation in the study. We estimated yields from 230 smallholder maize fields using alternative methods (Table 1). While the details of the yield estimation methods with schematic representation are presented in Kosmowski et al. (in press), the brief descriptions of the methods are as follows.

**Table 1:** Description of implemented yield estimation methods with the number of sampling points and sampling area. Sampling area is the mean with the range in the parenthesis.

Methods	Description	Sample points (n)	Category	Sampling area (m^2^)
M0	Farmers' prediction	1	Prediction	1610 (525–3750)
M1	MW path	10	Transect	10
M2	Horizontal transect	4	Transect	4
M3	Random quadrat	1	Crop Cut	16
M4	Three diagonal quadrats	3	Crop Cut	48
M5	Full plot harvest	8	Complete sample	1418 (416–2915)
M6	Random octant	1	Crop Cut	177(47–690)

#### 2.1.1 Farmer predictions (M0)

We asked farmers to predict the expected maize yield in quintals (100 kg units), while the maize was still unharvested. Farmers were also requested to estimate the area of the field in local units, after which we converted into meter square (m^2^). Maize grain yield was expressed in kilogram per hectare (kg/ha) using the two parameters.

#### 2.1.2 MW path (M1)

Enumerators identified the long side of the field. They, then, set an imaginary ‘M’ line starting at 1 m from the northwestern vertices of the fields and a ‘W’ line on the opposite long side. The ‘feet’ and the ‘vertices’ of the ‘M’ and ‘W’ lines were used as sampling loci, forming 10 sampling points. At each sampling loci, the enumerators randomly selected three cobs and recorded the number of cob-bearing plants within 1 m^2^ of the area surrounding the sampling points. The cobs were weighed unshelled, shelled and weighed, and the field moisture content of the grain was measured. We estimated yield in kg/ha using these parameters and plot area measured with high-accuracy total station theodolite. Grain yield was standardized at 12.5 % moisture content. We quantified intra-plot heterogeneity in stand density, cob population and grain yield, using the variability among the 10 sampling points within each plot/field.

#### 2.1.3 Horizontal transect (M2)

We identified the mid-point of the short side of the field using handheld GPS. We determined four sampling points at equal distances along a horizontal line that crosses to the mid-point of the opposite short side of the field. At each sampling point, we randomly selected and weighed three cobs. Enumerators recorded the number of cob-bearing plants within 1 m^2^ of the area surrounding the sampling points. The same procedure as in M1 was used to make yield estimation in kg/ha. We quantified intra-plot heterogeneity in stand density, cob population and grain yield, using the variability among the 4 sampling points.

#### 2.1.4 Random quadrat (M3)

A random quadrat was set using two random numbers within the length limits of the short and long sides of the field, starting at the Northwest corner of the field. At each point, a 4 by 4 m sample quadrat was defined. Within the 16 m^2^ quadrat, we quantified population density, cob-bearing proportion, and cob fresh weight. We randomly selected three cobs and recorded the fresh weight. We shelled grains from the middle part of the cobs and measured the field moisture content. The same procedure as in M1 and M2 above was followed to estimate dry grain yield in kg/ha. This method is the official method of yield estimation applied by the Central Statistics Agency (CSA) of Ethiopia.

#### 2.1.5 Diagonal quadrats (M4)

We identified the longest diagonal of the field starting from the northwestern vertex of the field. We set three equidistant sampling quadrats along the diagonal line. At each point, a 4 by 4 m sample quadrat was defined. Within the 16 m^2^ quadrats, we quantified population density, cob-bearing proportion, and cob fresh weight. We randomly selected three cobs and recorded their fresh weight. We shelled grains from the middle part of the cobs and measured the field moisture content. The same procedure as in M1 and M2 above was followed to estimate dry grain yield in kg/ha. We quantified intra-plot heterogeneity in stand density, cob population and grain yield, using the variability among the 3 sampling points.

#### 2.1.6 Full plot harvest (M5)

We harvested the entire maize in the field. Maize harvested in the previous methods was left at respective sampling points and included in the full plot yield measurement. In this method, the fields were divided into eight approximately equal-sized sub-plots. We measured the area of each sub-plot using the rope-and-compass method. We counted and recorded the fresh weight of all cobs within each of the subplots. Three cobs were selected randomly, weighed and shelled. We weighed the shelled grain and recorded its moisture content. The sample grain moisture content was used to adjust the final grain yield at 12.5 % moisture. Out of the eight sub-plots used for full plot harvest, we included a randomly selected octant as ‘random octant’ (M6) in further analysis, as a separate method. We used the full plot harvest as the ‘gold standard’ for yield estimation accuracy and true intra-plot heterogeneity. We computed the yield estimation errors from other methods against the baseline yield obtained from this method.

In further analysis and discussion, we categorized the above methods into transect methods (MW path and Horizontal transect), and crop cut methods (Random quadrat, Diagonal quadrat and the random octant). The full plot harvest method was categorized as ‘Complete sample’ method (Table 1).

### 2.2 Data analysis

#### 2.2.1 Heterogeneity

We identify two forms of heterogeneity. Inter-plot heterogeneity (i.e., heterogeneity between plots) is the variability in yield that occurs between different plots of farms. Intra-plot heterogeneity (i.e., within-plot or intra-field heterogeneity) is the variability in yield that occurs within a single plot or field of a farm when samples taken at distinct locations, extrapolated to the whole field, are computed. Throughout this paper, we use “plot” and “field” as synonyms.

We computed variability in crop stand density, cob density and grain yield, using the coefficient of variation (CV) as a measure of heterogeneity. To compute inter-plot variability, we first calculated the mean yield of each plot, for each of the methods described above. We then calculated CV for all fields per method. We applied Eq. 1 to compute inter-plot heterogeneity (variability among all plots for a method, using ordinary CV computation technique), as the sample size of 230 is relatively large, leading to less biased estimation of CV. Different CVs are expected for different methods as the methods generate samples from varying sample locations, number of replications, sampling area and plot orientation relative to the whole plot.

(1) $${CV}_{i}=\;100*\sqrt{\frac{\sum_{i}^{N}{\left(x_{i}-\;\mu_{i}\right)}^{2}}{{\mu_{i}}^{2}*(N-1)}}$$

where, *CV_i_* is the coefficient of variation between plots in the *i^th^* yield estimation method, *x_i_* is the i^th^ observation, and *μ*_i_ is the mean of the concerned variable using the *i^th^* yield estimation method.

Intra-plot heterogeneity is computed using the variabilities between samples for methods that had more than 2 sampling spots (MW path, horizontal transect and diagonal quadrats) and variabilities between subplots (eight sub-plots) in the full harvest method. In such small samples (3–8 sampling units), the ordinary coefficient of variation is possibly biased (Breunig, 2001). To minimize bias, we used the bias-corrected mean square method, suggested by Hyslop and White (2009) to compute CV. This technique helps to disentangle the magnitude of intra-plot heterogeneity, because any consecutive sample from a uniform population is expected to provide a constant yield when extrapolated for the whole area. By contrast, the existence of large variations in yields extrapolated from consecutive samples within a single field is an implication of internal variability (i.e., intra-plot heterogeneity), as computed by Eq. 2.

(2) $${CV}_{j}=100*\;\sqrt{\frac{\sum_{i}^{N}{\left(\lambda_{ik}/\alpha_{ik}\right)}^{2}}{{2N}_{j}}}$$

*CV_j_* is the intra-plot heterogeneity within the j^th^ plot, $\lambda_{ik}$ is the difference between two paired measurements i and j, ${\text{α}}_{ik}$ is the mean of paired measurements, i and k, and Nj is the total number of samples or data pairs within the j^th^ method. We mainly used the intra-plot heterogeneity in grain yield for further analysis. We estimated the total variability through the weighted combination of inter- and intra-plot variabilities as outlined in (Lee et al., 2002). We, then, calculated the proportion of each variability against this estimated total variation.

#### 2.2.2 Probabilities of accurate yield estimation

Different yield estimation methods tend to be prone to different levels of estimation errors. For a method that estimates the yield accurately, the mean of the error approximates to zero and is expected to have a small variance around the mean. Assuming a normal distribution for the estimation error in each method, the probability for a method to estimate yield within a certain range of accuracy can be computed by using Eq. 3 (Spiegel et al., 2013).

(3) $$\begin{array}{cc} P\left[l_{i}<\;x_{i}<\;u_{i}\right]= & \int_{l_{i}}^{u_{i}}\frac{1}{\sqrt{2\pi v_{i}}}*\;e^{-\frac{1}{{2v}_{i}}{\left(x_{i}-\;m_{i}\right)}^{2}}\delta x_{i} \end{array}$$

where,

P = the probability of estimated yield falling between $l_{i}$ and ${\;u}_{i}$,

$l_{i}$ = the lower bound of the error value above which the probability is to be computed,

$u_{i}$ = the upper bound of the error value below which the probability is to be computed,

$x_{i}$ = the i^th^ deviation (estimation error) from the true yield obtained from full plot harvest,

$v_{i}$ = the variance of the distribution for the *i^th^* yield estimation method and

$m_{i}$ = the mean error for the *i^th^* yield estimation method

#### 2.2.3 Intra-plot heterogeneity and yield estimation errors

We used a generalized linear mixed model (GLMM) to explore the association between intra-plot heterogeneity and the yield estimation accuracy of a method. The standardized error, which is the magnitude of yield deviation regardless of whether the deviation lies above or below the true yield measured from the full plot, was used as a dependent variable (Eq. 4).

(4) $$\varepsilon_{ij}=\left(\frac{\left|\mu_{i}-x_{j}\right|}{\mu_{i}}\right)*100$$

where $\varepsilon_{ij}$ is the magnitude of the error in the *j^th^* method expressed as a percentage of the true yield measured corresponding to the *i^th^* full plot, $\mu_{i}$ is the *i^th^* observation of yield measured through full plot harvest, $x_{j}$ is the *j^th^* observation of yield estimation corresponding to the true yield measurement $\mu_{i}$. We used the intra-plot heterogeneity computed in Eq. 2, the yield estimation methods (including one random octant out of the eight sub-plots as separate method) and the interactions between yield estimation methods and heterogeneity as independent variables to predict the standardized yield estimation error. We modeled these two variables as fixed effects, allowing random effects at the *woreda* (i.e., district) level (Eq. 5).

(5) $$\varepsilon_{ij\left(k\right)\;}=\;\alpha +\pi W_{k}+\;\beta M_{i\left(k\right)}+\;\eta H_{j(k)}+\;\lambda \;{MH}_{ij\left(k\right)}\;\;+\;R$$

Where $\varepsilon_{ij\left(k\right)\;}$ is the standardized error from the *i^th^* method and j^th^ intra-plot heterogeneity nested in the *k^th^* woreda, $M_{i\left(k\right)\;}$ is the *i^th^* yield estimation method nested in the *k^th^* woreda, $H_{j\left(k\right)\;}$ is the *j^th^* heterogeneity nested in the k^th^ woreda, ${\text{W}}_{\text{k}}$ is the k^th^ woreda and R is the residual of the regression. ${\text{M}\text{H}}_{\text{i}\text{j}\left(\text{k}\right)}$ is the interaction between the standardized error from the *i^th^* method and j^th^ intra-plot heterogeneity nested within the k^th^ *woreda.* Constants α, π, β, η, and λ are coefficients for the fixed and random effects of the regression and their interactions.

#### 2.2.4 Intra-plot heterogeneity and farm intensification

In addition to natural plot characteristics that drive heterogeneity in smallholder systems (Tittonell et al., 2013), intra-plot heterogeneity can be triggered by different socio-economic settings of the households, input intensity and past and current agronomic practices. We applied a parsimonious variable selection procedure of the forward Akaike information criterion (AIC) algorithm to identify variables that best correlate to the intra-plot heterogeneity (Table S1). This algorithm selects variables that minimize error and improve the general fit of the model. The forward elimination algorithm converged after 62 iterations (at the lowest possible AIC = 1479, R = 0.74, RMSE = 7.50, R^2^ = 0.55, MSE = 56.28, and MAE = 4.94). We fitted a GLMM with the selected variables as specified in Eq. 6.

(6) $$Y_{ijl\left(k\right)\;}=\;\alpha +\pi W_{k}+\;\beta X_{i\left(k\right)}+\;\eta P_{j(k)}+\;\;\text{μ}S_{l(k)}\;\;+\;\lambda \;{XH}_{ij\left(k\right)}\;\;+\;\text{τ}\;{XS}_{il\left(k\right)}\;+\;\text{σ}\;{HS}_{jl\left(k\right)}\;\;+\;\;\varepsilon_{ijl(k)}\;$$

where ${\text{Y}}_{\text{i}\text{j}\text{l}\left(\text{k}\right)\;}$ is intra-plot heterogeneity in maize yield, $\;{\text{X}}_{\text{i}}$ is the *i^th^* vector of input variables, ${\text{P}}_{\text{j}}$ is the *j^th^* vector of management practices and ${\text{S}}_{\text{l}}$ is the *l^th^* vector of socio-economic factors and ${\text{W}}_{\text{k}}$ is the k^th^ woreda. ${\text{X}\text{H}}_{\text{i}\text{j}\left(\text{k}\right)}$, ${\text{X}\text{S}}_{\text{i}\text{l}\left(\text{k}\right)}$ and ${\text{H}\text{S}}_{\text{j}\text{l}\left(\text{k}\right)}$, respectively, are interactions between vectors of i^th^ input and j^th^ management variables, i^th^ input and l^th^ socio-economic factors, and j^th^ management and l^th^ socio-economic factors; all nested in k^th^ woreda, while ε is the residual of the regression. α, π, β, η, μ, λ, τ and σ are regression coefficients. The vector of input variables (X_i_) such as type of maize variety, seed rate, rates of Urea and NPS fertilizers, manure, pesticide and herbicide application; the vector of management practices (*P_j_*) such as time of planting, time of fertilizer application, permanent soil conservation, inter-row and inter-plant spacing and level of plant damage in the plots; and the vector of socio-economic factors *(S_l_*) such as household and hired labor, the distance of the plot from home, market and the main road, were selected to be important through the AIC algorithm. Biophysical variables suspected to affect intra-plot heterogeneity such as slope, landscape position, plot area, physical structures and presence of trees within the field were dropped during the variable selection algorithm. While the input, management and socio-economic variables were all modeled as fixed effects, woreda was modeled as a random effect. We removed higher degree interactions (i.e., three-way interactions and above) from the final model to avoid computational tractability and interpretation complexity.

For all regressions, we used the probability level of 0.05 to test significance, unless otherwise stated. Interactions and main effects that had small explanatory power, i.e., variables with F-values lower than 0.1, were dropped from the final model.

## 3 Results

### 3.1 Inter-plot and intra-plot heterogeneity

**Fig. 2:**
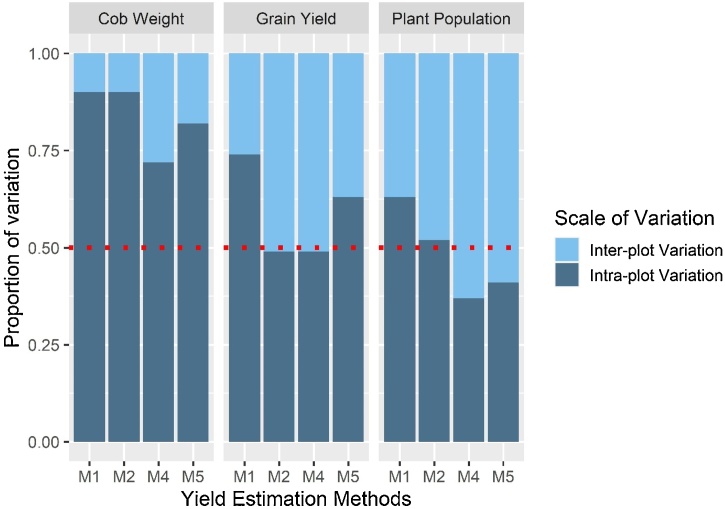
Proportion of inter- and intra-plot variability in maize population density, cob weight and grain yield following different sampling designs. The dashed horizontal line indicates 50 % share of inter- and intra-plot heterogeneity.

Table 2 presents the levels of between field variability in maize population density, cob weight and grain yield as captured by different yield estimation protocols. Expectedly, large variability exists between fields of smallholder farmers regardless of the method used (32–56 %, 22–73 % and 39–51 % variation in population density, cob weight and grain yield, respectively). Comparing the random quadrat, the diagonal quadrat and the full plot harvest methods for their inter-plot variability, it seems an increase in the number of crop cutting units (quadrats) reduces variability slightly for cob and grain yield (Table 2). On the other hand, inter-plot variability in grain yield from the random octant is similar to other methods, while variability for cob and density was much higher. In general, transect methods (MW path and Horizontal transect) captured more intra-plot variability than crop cut methods (random quadrat and diagonal quadrat), and the intra-plot variability was higher for cob weight than grain yield or plant population (Fig. 2).

**Table 2:** Levels of between fields mean variability captured by different yield estimation (sampling) methods. CV = Coefficient of variation (%). M6 = the random octant.

	Between fields variability (% CV)		
Methods	Population density	Cob Weight	Yield
M0	–	–	45.5
M1	35.9	25.1	47.7
M2	36.6	28.2	51.0
M3	36.8	30.2	48.6
M4	32.5	24.1	40.3
M5	49.3	22.0	39.0
M6	55.5	64.2	43.4

In summary, the random octant captured a significantly larger share of variability for maize yield components (population density and cob weight) among fields compared with any of the commonly implemented crop yield estimation methods.

Different yield estimation methods show different levels of bias in capturing inter- and intra-plot heterogeneity in maize yield and yield components (Fig. 2). For most methods and most measured variables, intra-plot variation is larger than inter-plot variation. The coefficient of variation for intra-plot heterogeneity ranged from 1 to 99 %, depending on the type of yield estimation method used (Table S 2). For maize grain yield, which is the ultimate measure of productivity, the horizontal transect (M2) and diagonal quadrats (M4) protocols appear to overlook intra-plot heterogeneity and amplify inter-plot heterogeneity compared with the full harvest method (M5, which is assumed to have captured approximately all the inter- and intra-plot variations). By contrast, the MW path (M1) method seems to amplify intra-plot heterogeneity over inter-plot heterogeneity compared with the values under full harvest.

**Fig. 3:**
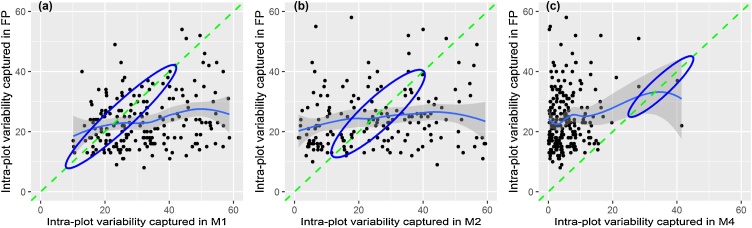
The proportions of variability captured by the MW path (a), horizontal transect (b) and three sets of diagonally oriented quadrats method (c) methods as compared with the variability captured in full plot harvest. The dashed line indicates a 1:1 relationship, while the ellipse encircles points that are equidistant from the fitted and 1:1 line.

As shown in Fig. 2, all three methods (excluding the full plot harvest) captured significant portions of intra-plot variation. For some variables, they even appeared to reveal more intra-plot variation than the full plot harvest method. Fig. 3 explores how the variabilities captured by those methods compare to the variabilities captured in full plot harvest, assuming the full plot harvest captures most of the intra-plot variability in grain yield. In general, the three different methods did not capture intra-plot variability similarly. For example, the MW path (Fig. 3 a) shows a tendency of amplifying extremely small intra-plot variability (most points lie below the 1:1 line), contrasting with the diagonal quadrats method (Fig. 3 c). This observation is sensible as the MW path samples close to the borders, where intra-plot variability is expected to be the highest, while diagonal quadrats samples through the center of the plot, where intra-plot variability is expected to be lower.

Assuming the full plot harvest captures the true intra-plot variability in grain yield (i.e., ∼100 variability), the combination of the three methods (i.e., if we merge all the samples and extrapolate grain yield to the whole area) captures about 77 % of the total intra-plot variability. The MW path method (Fig. 3 a) alone captures 56 % of the true intra-plot variability (points lying within the ellipse) compared with the full plot harvest method. The horizontal transect method (Fig. 3 b) captures only about 19 % of the true intra-plot variability (points lying within the ellipse), while the diagonal quadrats method captured less than 1% of the true intra-plot variability in maize grain yield.

### 3.2 Distribution of yield estimation errors

**Fig. 4:**
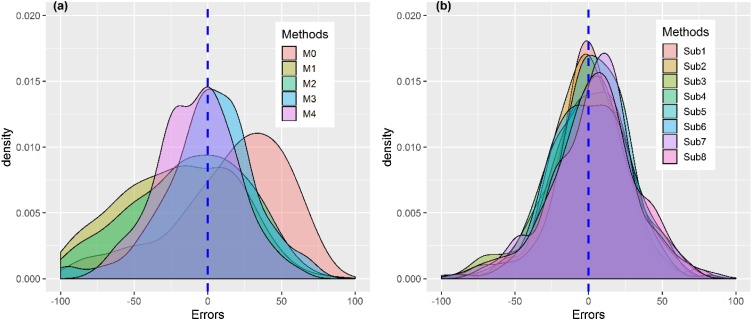
The distribution of yield estimation errors across conventional methods (a) and the sub-plots (b). The vertical dotted line indicates no error.

Yield estimation errors, the scaled deviation of yields from the true measure (full plot yield), appear to be more scattered away from the true yield for most of the conventional methods (Fig. 4 a), while the errors are concentrated around the expected mean error of zero in the sub-plots (Fig. 4 b). In Fig. 4 a, farmers’ prediction appears to underestimate maize grain yield compared with the transect methods (MW and horizontal transects), which tended to overestimate maize grain yield compared with the true yield obtained from full plot harvest. The crop cut methods, i.e., random quadrat and diagonal quadrats methods, seem to capture the true yield, as the mean errors are concentrated around zero (Fig. 4 a). By contrast, most of the sub-plots reduced mean error with the errors centering around zero (Fig. 4 b).

For farmers’ prediction, the MW path and random quadrat estimates, the probability of a yield estimate falling within 10 % of the true yield is less than 20 % (Table 3). Random quadrat and diagonal quadrats methods, respectively, are 25 % and 28 % probable to estimate the yield within the range of 10 % of the true yield. Except for the three diagonal quadrats protocol, which estimated the yield with the probability of 53 % to lie within 20 % of the true yield, all the other conventional methods estimated the yield to lie outside the 20 % range of the true mean.

**Table 3:** Probabilities of an estimated yield to be within 10 and 20 % of the true yield (yield from full plot harvest).

	Probabilities of an estimated yield to lie within	
Methods	10 % of the true yield	20 % of the true yield
M0	0.187	0.363
M1	0.178	0.347
M2	0.195	0.379
M3	0.252	0.479
M4	0.284	0.533
M6	0.298	0.556

By contrast, the yields estimated from the random octant (M6) had almost a 30 % probability of falling within 10 % of the true yield. For all yield estimations from the sub-plots, the yields were more than 50 % likely to fall within 20 % of the true yield.

For all conventional methods (M0-M4), we observed a highly significant (*P < 0.001*) main effect of yield estimation methods on the magnitude of standardized relative error (Table 4). The subplot-based yield estimations (i.e., random octant/M6) had a non-significant effect on the magnitude of standardized relative error at a 5% probability level. The magnitudes of the effects from the subplots (not reported here) are very small, indicating any of the subplots estimated yields closer to the true value compared with the other methods.

**Table 4:** Effect of intra-plot heterogeneity and yield estimation methods on the deviation of estimated yield from the true yield. Probabilities indicated in bold represent the significance of the effect at 5% probability level. SE = Standard Error.

Variables	Effects	SE	t-value	Pr(>|t|)
Main effect (Methods)				
M0	42.7	3.3	13.1	**0.0000**
M1	22.5	3.4	6.6	**0.0000**
M2	26.4	3.4	7.8	**0.0000**
M3	15.2	3.2	4.8	**0.0000**
M4	14.5	3.2	4.5	**0.0000**
M6	0.99	3.2	0.3	0.7596

Main effect (Heterogeneity/CV)				
Intra-plot CV	−0.29	0.13	−2.27	**0.0236**

Interaction effects (Methods * CV)				
M1 : Intra-plot CV	0.81	0.18	4.42	**0.0000**
M2 : Intra-plot CV	0.53	0.18	2.89	**0.0039**
M3 : Intra-plot CV	0.63	0.18	3.59	**0.0003**
M4 : Intra-plot CV	0.59	0.18	3.35	**0.0008**
M6 : Intra-plot CV	1.14	0.18	6.44	**0.0000**

The size of intra-plot heterogeneity also showed a negative and significant (*P < 0.05*) main effect on the magnitude of standardized relative error, underlining errors increase with a method’s failure to capture heterogeneity accurately. More importantly, the interaction effect between the yield estimation protocols and the size of intra-plot heterogeneity was highly significant (*P < 0.001*) for all methods, indicating that the effect of heterogeneity on the magnitude of standardized relative error is method-dependent.

From the interaction effects in Table 4, there is a 1% increase in estimation error from the MW path, horizontal transect, random quadrat and diagonal quadrats methods for every 0.8 %, 0.5 %, 0.6 % and 0.6 %, increase in intra-plot heterogeneity, respectively. Although these effects are small in magnitude, they are highly statistically significant (*P < 0.001*). The outcome of the interaction effect is similar for the random octant (M6) and the other sub-plots (not presented here).

In summary, the transect-based methods predict yields less accurately than the quadrat-based methods. The probability that these methods predict yields within the 20 % of the true yield (as predicted from the full plot harvest) is small (less than 0.5 in most cases). In addition, yield estimation errors were method dependent.

### 3.3 Farm intensification, plot accessibility and intra-plot heterogeneity

#### 3.3.1 How do input decisions affect intra-plot heterogeneity in yield?

Type of maize variety, seed rate, rate and timing of urea and NPS fertilizers, pesticide and herbicide application were important agronomic decisions affecting intra-plot heterogeneity in smallholder maize systems (Table 5). For every 0.2 kg/ha increase in the rate of urea, intra-plot heterogeneity in maize yield decreases significantly (*P < 0.05*). Basal (at planting) and top-dressing (split application) of this fertilizer decrease intra-plot heterogeneity even more significantly.

**Table 5:** Summary of the result of a regression model showing the variation in intra-plot heterogeneity in smallholder maize as a function of input, management and socio-economic proxies of intensification. Probabilities with significant effects (P < 0.05) are indicated in bold. Probabilities with significant effects at 10 % probability level are presented in bold. SE = Standard Error.

Variables	Effects*	SE	t-value	Pr(>|t|)
Main effects				
(Intercept)	17.6	13.9	1.3	0.2063
Seed rate	−0.5	0.4	−1.1	0.2627
Urea rate	−0.2	0.1	−2.9	**0.0041**
Urea at planting (Yes)	−30.1	11.6	−2.6	**0.0106**
Herbicide (Yes)	−0.5	1.8	−0.3	0.7742
Urea split application (Yes)	−15.8	4.6	−3.4	**0.0007**
Distance to home	−0.02	0.05	−0.4	0.6796
Distance to market	−0.1	0.02	−2.5	**0.0139**
Distance to main road	−0.1	0.04	−2.1	**0.0382**

Interaction effects				
Herbicide (Yes) x Variety (Local)	−7.4	8.5	−0.9	0.3853
Seed rate x Pesticide (Yes)	−1.1	0.5	−2.3	**0.0202**
Seed rate x Urea split application (Yes)	−1.1	0.5	−2.1	**0.0394**
Seed rate x NPS rate	−0.1	0.03	−2.8	**0.0060**
Pesticide (Yes) x NPS rate	0.1	0.1	1.5	0.1489
NPS rate x Urea at planting (Yes)	0.1	0.04	3.5	**0.0007**
Urea rate x Pesticide (Yes)	−0.1	0.1	−2.2	**0.0300**
Pesticide (Yes) x Urea at planting (Yes)	−13.2	6.5	−2.0	**0.0447**
Herbicide (Yes) x Urea at planting (Yes)	−6.8	4.2	−1.6	0.1076
Herbicide (Yes) x Pesticide (Yes)	−6.8	5.0	−1.4	0.1757
Pesticide (Yes) x Permanent conservation (Yes)	18.9	6.3	3.0	**0.0031**
Pesticide (Yes) x Spacing factor	−104.9	48.2	−2.2	**0.0307**
Pesticide (Yes) x Inter-row spacing	1.0	0.3	3.2	**0.0015**
Permanent conservation (Yes) x Inter-Plant spacing	0.3	0.3	0.9	0.3590
Inter-Plant spacing x Late planting	0.02	0.04	0.4	0.6769
Inter-Plant spacing x Medium planting	0.03	0.04	0.7	0.5088
Early planting x Observable damage (Yes)	10.0	10.4	1.0	0.3391
Late planting x Observable damage (Yes)	23.7	7.9	3.0	**0.0031**
Medium planting x Observable damage (Yes)	18.4	6.4	2.9	**0.0045**
Spacing factor x Observable damage (Yes)	74.7	33.9	2.2	**0.0288**
Inter-plant spacing x Observable damage (Yes)	−0.8	0.3	−2.7	**0.0082**

* Average marginal effects (AME) of each variable is presented in Table S3.

More importantly, these agronomic decisions interacted with one another in affecting intra-plot yield heterogeneity. Higher seed rates combined with pesticide, urea top dressing and higher NPS rates decrease intra-plot heterogeneity significantly (*P < 0.05*). Significant (*P < 0.05*) declining trend in intra-plot heterogeneity was observed when a higher rate of urea was combined with pesticides. In addition, high rate of urea applied at planting significantly reduced intra-plot heterogeneity (*P < 0.05*). By contrast, a higher rate of NPS combined with pesticides and urea at planting was found to increase intra-plot heterogeneity significantly (*P < 0.05*). Higher inter-row spacing, which is a measure of stand density in maize, increases intra-plot heterogeneity, regardless of pesticide application, indicating the application of pesticide on sparsely populated fields does not improve intra-plot heterogeneity. Unexpectedly, pesticide applied on well conserved fields affect intra-plot heterogeneity significantly (*P < 0.05*). On late and medium planted fields and with observable plant damages, intra-plot heterogeneity was significantly high (*P < 0.05*). Similarly, on plots with a higher spacing factor, which is a multiple of inter-row and inter-plant spaces, and higher observable plant damages, intra-plot heterogeneity was significantly higher.

#### 3.3.2 How do agronomic decisions affect intra-plot heterogeneity in maize yield?

Agronomic management practices such as time of planting, time of fertilizer application, permanent soil conservation; inter-row and inter-plant spacing and crop damage in the fields affected the magnitude of intra-plot heterogeneity significantly (Table 5). Both basal and top dressing of urea had a similar and significant (*P < 0.05*) effect of lowering intra-plot heterogeneity in maize yield. However, the magnitude of the effect was more pronounced for application at planting (30.1 %) than split application (15.8 %). Sparse plant density as observed from higher spacing factor and higher inter-plant spacing significantly (*P < 0.05*) increased intra-plot heterogeneity in plots where significant observable damage was recorded. Late planted plots that showed higher observable damages were significantly (*P < 0.05*) heterogeneous within the plot.

#### 3.3.3 How does plot accessibility affect intra-plot heterogeneity in yield?

Plot accessibility factors such as the distance of the plot from home, market and main road significantly affected intra-plot heterogeneity (Table 5). For example, plots located near main market centers and asphalt roads showed significantly (*P < 0.05*) higher intra-plot heterogeneity than plots that are far away from these access facilities. The accessibility factors also interacted in affecting intra-plot heterogeneity.

#### 3.3.4 Intra-plot heterogeneity and selected outcomes of intensification

**Fig. 5:**
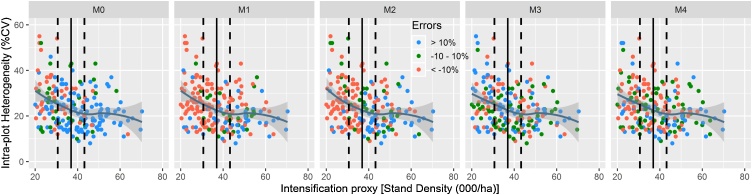
Stand density as an indicator of output intensification and its relationship with intra-plot heterogeneity across yield estimation methods. The bold vertical lines indicate the mean, while the broken vertical lines are 0.5 standard deviations away from the mean in both directions.

We considered grain yield and stand density as proxies of intensification among output variables. For all yield estimation methods, intra-plot heterogeneity appears to decline with higher stand density (Fig. 5). The intra-plot heterogeneity stabilizes at about 0.5 standard deviations of the mean. Whether the method overestimates, underestimates or estimates closer to the true yield was found to depend on the stand density and intra-plot heterogeneity. For example, MW path and horizontal transect methods generally overestimate (errors < -10 %) yield and more so under low stand density and higher intra-plot heterogeneity. While farmers’ prediction underestimates yields, in average, it showed a tendency to overestimate yields at lower stand density and higher intra-plot heterogeneity. The random and diagonal quadrat protocols estimated the yield relatively closer to the true yield. Both the accuracy and errors incurred in the latter two methods are independent of the stand density and intra-plot heterogeneity, as overestimations, underestimations and correct estimations were evenly distributed across all densities and heterogeneity levels.

**Fig. 6:**
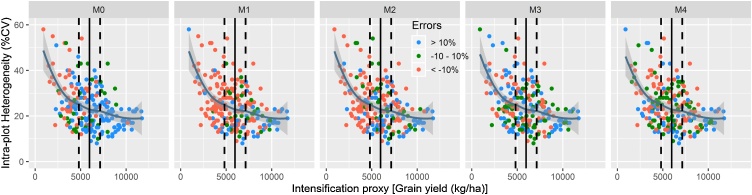
Maize grain yield as an indicator of output intensification and its relationship with intra-plot heterogeneity across yield estimation methods. The bold vertical lines indicate the mean, while the broken vertical lines are 0.5 standard deviations away from the mean in both directions.

Fig. 6 shows that intra-plot heterogeneity in maize grain yield decreases with higher yields regardless of the yield estimation method used. However, the sharp decline in intra-plot heterogeneity is apparently more pronounced for yields that are below the mean grain yield for all methods. Yield overestimation (with errors of < -10 %) appears to dominate low-yielding fields (i.e, fields with grain yield less than the mean). For farmers’ predictions, MW path and horizontal transect methods, the overestimations mainly occur for plots yielding lower than 0.5 standard deviation of the mean. In these methods, underestimation is dominant for plots yielding above the mean. For random and diagonal quadrats, there is no clear distinction between overestimation and underestimation relative to the ±10 % marks of the mean. For all methods, relatively accurate estimations (within ±10 % error margins) occur highly concentrated between 0.5 standard deviations of the mean.

**Fig. 7:**
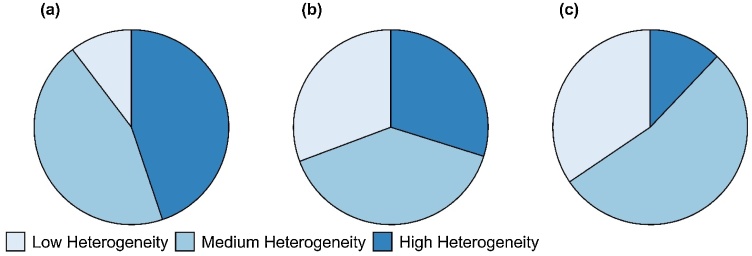
The distribution of intra-plot heterogeneity across low-yielding (a), medium-yielding (b) and high-yielding (c) plots. Low- and high-yielding plots represent plots with grain yields lower than the first quartile and higher than the third quartile, respectively, while medium-yielding plots are found in between the two. Similar classification was used for distributions of heterogeneity in yield.

Only 10 % of the low-yielding plots (Fig. 7 a) show intra-plot heterogeneities falling lower than the first quartile. Most low-yielding plots show either medium or high intra-plot heterogeneity. The proportions of internally most heterogeneous plots (i.e., plots falling above the third quartile for their intra-plot heterogeneity) decline from 30.7 % in medium-yielding fields (Fig. 7 b) to 12.1 % in high-yielding ones Fig. 7 c). Intermediate intra-plot heterogeneity is almost equally distributed across all levels of plot productivity (Fig. 7).

## 4 Discussion

### 4.1 Intra-plot heterogeneity is significant and larger than inter-plot heterogeneity

Our results show the existence of high heterogeneity between fields, as evidenced by the coefficient of variation as high as 73 %, for example in cob weight. The heterogeneity in grain yield was similarly high, with a coefficient of variation ranging from 39 to 51%. Such extreme heterogeneities in maize yield among smallholder farms are not new. Using CV, Tittonell et al. (2007) reported up to 60 % variability among smallholder farms in Kenya. Similarly, Ichami et al. (2020) showed between farm heterogeneity can range from 50 to 89% in smallholder systems, attributing 31 % of the variation to soil properties. Although significant heterogeneity has been documented in many African smallholder production systems, agronomic research for development generally ignores such heterogeneity in the formulation of productivity-boosting recommendations and programs (Vanlauwe et al., 2019).

Interestingly, we find heterogeneity in maize grain yield between farms to vary with the yield estimation method used (Table 2). Assuming the heterogeneity captured in the full plot method as the best predictor of true heterogeneity, most yield estimation methods appear to fail at capturing true heterogeneity in maize grain yield between smallholder fields. Farmers’ predictions, the MW path, horizontal transect, random quadrat, and diagonal quadrat methods, respectively, amplified between-plot heterogeneity by 17, 23, 31, 25, 3%, compared with the between-plot heterogeneity estimated using the full plot method.

Also of interest is our finding that within-field heterogeneity is more dominant than between-field heterogeneity in smallholder maize yield (Fig. 2). Again, the proportion of within- and between-field heterogeneity compared with the total heterogeneity was method-dependent (Fig. 3). For horizontal transect and diagonal quadrats methods, intra-plot variability constitutes almost 50 % of the total variability in maize yield. In full plot and MW path methods, intra-plot variability constituted 62 and 74 %, respectively, of the total variability observed, underlining intra-plot heterogeneity can be more dominant than inter-plot heterogeneity in these systems. This implies the largest proportion of the total variability arises from large internal variability (intra-plot) compared with the variabilities between plots (inter-plot). Between field heterogeneity was already deemed challenging for accurate yield estimation even from the much-preferred crop cuts (Chivasa et al., 2017; Joshi et al., 2019; Ngoune Tandzi and Mutengwa, 2020), efficient soil fertility management (Boydell and McBratney, 2002; Tittonell et al., 2007), tailored sustainable intensification options (Falconnier et al., 2016), proper understanding of the risk of technology success or failure (Vanlauwe et al., 2019) and site-specific soil nutrient management (Vasu et al., 2020). Using zones of stable heterogeneity (Khitrov et al., 2019), farm typologies (Tittonell et al., 2007), precision agriculture (Patzold et al., 2008), site-specific soil fertility management (Vasu et al., 2020) and application of non-average metrics to better understand smallholder systems (Vanlauwe et al., 2019) were suggested, among other approaches, to deal with between field heterogeneity inherent in smallholder cropping systems.

By contrast, intra-plot heterogeneity has been overlooked. Understanding and quantifying intra-plot heterogeneity is more difficult than identifying and measuring inter-plot heterogeneity. Operationalizing intra-plot heterogeneity in agronomic decision-making is even more challenging (Vanlauwe et al., 2019). However, understanding, quantifying, and managing intra-plot heterogeneity is needed to improve resource use efficiency, land use planning, accurate yield measurement, precision farming and site-specific nutrient management.

### 4.2 Yield estimation methods that fail to capture true intra-plot heterogeneity are less accurate

Fig. 3 shows the absence of a strong correlation between the intra-plot heterogeneities captured by conventional methods (MW path, horizontal transect, and diagonal quadrats) and the full plot method, which is considered the best estimator of true intra-plot heterogeneity. There is an established assumption that yield estimation methods introduce errors because sampling does not fully represent the true nature of the population (Gourlay, 2019). Most sampling designs fail to capture the true variability that exists in the population of interest (Jin et al., 2017). Our findings hint that sampling methods that capture larger heterogeneity than actually existed could also be problematic. For example, even the method capturing the largest intra-plot heterogeneity (MW path) was found to cover only 56 % of the true variability. However, the MW path method is not the most accurate yield estimator (Fig. 4), indicating that capturing maximum intra-plot heterogeneity alone does not guarantee maximum method accuracy. While Fermont and Benson (2011) reported sampling methods that focus on uniform spots to be associated with larger estimation errors arising from systematic and non-systematic biases, our findings underscore that the opposite is also true. Spatially diffuse sampling methods (hence, likely to introduce too much heterogeneity e.g., MW path, which samples across the plot margins in the MW shape) are error prone. As a result, methods that are spatially concentrated, as well as methods that are very spatially diffused (e.g. transects) perform less accurately than methods that balance concentration and diffusion of sampling within a plot (e.g., diagonal quadrats).

The magnitude (Table 4) and distribution (Fig. 4) of yield estimation errors are method-dependent. For all conventional methods, except the random quadrat and diagonal quadrat methods, the errors are skewed left, indicating the dominance of yield overestimation problem under these methods (Fig. 4 a). The probabilities that these methods (farmers’ predictions, MW transect and horizontal transect) estimate yield within 10 % of the true yield were less than 0.2 (Table 3), showing that their error margins are larger than 10 % for 80 % of the time. Even the most accurate methods (random and diagonal transects) estimated yield within 20 % of the true yield only half of the time. Such high probabilities of mis-estimation have great management and policy implications, especially as these errors possibly accumulate at larger scales such as national level. For example, a study that uses farmers’ yield estimation from a similar study area, Assefa et al. (2020) found an average maize yield of 2.9 t/ha. Applying the estimation accuracy of this method from the current work, the probability of this yield falling within 50 % of the true yield becomes about 0.5. Similar estimation probability can be applied to the reported yield gaps from this area. As the method-dependent plot level estimation errors tend to aggregate along scales (i.e., from the field to national) because of their additive properties (Colombi et al., 2014), policy makers need to handle the yield values from these methods with caution (Sapkota et al., 2016).

By contrast, subplot-based yield estimations show improved estimation accuracy (Fig. 4 b), although these methods are expensive and time-consuming. Combinations of the conventional methods with sub-plots possibly improve estimation accuracy significantly within a reasonable time and affordable cost. For example, random placing of the variants of horizontal transect within each of the subplots could improve estimation accuracy. On the other hand, the estimation accuracy of the methods varied with intra-plot heterogeneity (Table 4). For example, in the worst-performing method (horizontal transect), only a minor increase in intra-plot heterogeneity (∼0.5 %) causes a significant increase in the estimation error when this method is used to estimate maize yield. Methods that capture less intra-plot heterogeneity (by ∼0.3 %) incur more yield estimation errors (by 1%) over the baseline. While our work identifies the ‘best-performing’ estimation method under inherently heterogeneous systems, designing accurate universal yield estimation under systems exhibiting considerable heterogeneity remains extremely challenging (Jin et al., 2017). A pragmatic, case-by-case approach tailored to specific cropping system properties, is suggested (Hoefsloot et al., 2012).

### 4.3 Intra-plot heterogeneity is correlated with variations in agronomy, input intensity and plot accessibility factors

We found that measures of agricultural intensification, poor agronomy, plot accessibility factors and their interactions affect intra-plot heterogeneity in smallholder maize systems in Ethiopia (Table 5). This suggests agricultural extensification, which is also a driver of low yield in smallholder maize systems (Dubey et al., 2020), drives intra-plot heterogeneity in these systems. As reported by Tilman et al. (2011) increased inputs, improved agronomic practices and improved crop varieties are required to achieve higher crop yields. Our results show that low fertilizer rates, inappropriate time of fertilization, poor crop protection and use of unimproved crop varieties increase intra-plot heterogeneity. Variation in soil management and fertility-enhancing inputs has long been established to cause heterogeneity in smallholder farms (Patzold et al., 2008; Tittonell et al., 2013, 2007). We also found intra-plot heterogeneity reduces yield estimation accuracy, regardless of the sampling method followed, although the magnitude of estimation error was method-dependent. Thus, optimum input and appropriate agronomy have a double advantage of improving smallholder crop productivity, which is a huge goal in itself, and reducing intra-plot heterogeneity, which improves accuracies of yield estimation in these systems.

The decline in intra-plot heterogeneity with increasing plant population (Fig. 5) and grain yield Fig. 6), affirms that agricultural intensification decreases intra-plot heterogeneity in these systems. Low-yielding plots, in average, tended to overestimate, while high-yielding plots tended to under-estimate yields when combined with large intra-plot heterogeneity. Visual inspections that aid to guesstimate whether the field is low- or high-yielding may help to decide yield estimation method with optimum accuracy, under existing circumstances. In essence, different protocols could be developed based on their performance in cropping systems with particular heterogeneity. While such approaches maybe applied as a long-term plan, they are difficult to implement under many smallholder circumstances

### 4.4 Limitations and way forward

Although we have identified the magnitudes and distributions of inter- and intra-plot heterogeneity in smallholder maize systems and the method-dependence of yield estimation accuracy under inherently heterogeneous systems, a couple of questions remain unaddressed in this paper, mainly because of experimental design and data limitations. For example, we have clearly shown that yield estimation accuracy deteriorates with rising intra-plot heterogeneity, although our data and approach do not enable us to clearly propose how to improve yield estimation accuracy under such circumstances. Future works may apply stochastic predictive simulation modeling to identify approaches that improve the method-dependent yield estimation accuracy under these heterogeneous systems. We were unable to disentangle the root drivers of some relationships in this work because of data limitations such as type, the active ingredient, concentration and rate of pesticides and herbicides explored in our study. Lack of data on soil parameters, which has been identified as a key driver of yield heterogeneity in smallholder systems (Tittonell et al., 2013), has limited the scope of our analysis.

Furthermore, this work could not identify if there exists an appropriate level of heterogeneity that minimizes yield estimation errors for all methods. Because heterogeneity is an inevitable property of smallholder systems (Tittonell et al., 2013), understanding the level of heterogeneity that minimizes method-dependent yield estimation error becomes important. How method- and heterogeneity-induced yield estimation inaccuracies aggregate at scales bigger than the plot (such as district, zone, state and national) could be important points for future exploration. Such explorations assist national food security and yield gap management decisions.

## 5 Conclusions

Intra-plot heterogeneity is an inherent property of smallholder systems in sub-Saharan Africa, although there has been relatively little research on its drivers or its implications. Our goals have been to explore the magnitude of inter- and intra-plot heterogeneity, to describe the relationship between intra-plot heterogeneity and land productivity and disentangle its impact on the accuracy of yield estimation protocols in smallholder maize systems. Using well-measured data from 230 smallholder maize fields in Ethiopia, we conducted full plot harvests in regular plot sub-divisions to measure yields and yield variability. While intra-plot heterogeneity is more dominant than inter-plot heterogeneity, both types of variabilities are conspicuous in these systems. We find elevated levels of intra-plot variability in stand population, cob weight and grain yield. This heterogeneity is associated with low levels of intensification, i.e. more intensively managed plots have more internally homogeneous productivity. In accordance with sampling theory, we find that this internal heterogeneity in plot productivity has implications for yield estimation strategies: sampling methods which better capture internal heterogeneity are more accurate.

The magnitude and distribution of yield estimation errors varied depending on the degree of intra-plot heterogeneity in maize. However, capturing large intra-plot heterogeneity alone does not guarantee maximum method accuracy, as methods that amplified heterogeneity were similarly erroneous.

Incorporating heterogeneity in decision-making to minimize its effect requires systematic quantification of heterogeneity, firsthand. Nonetheless, quantifying intra-plot heterogeneity has been a big challenge. Our results imply that yield measurement strategies in smallholder production systems should be defined in ways that acknowledge known patterns of intra-plot heterogeneity in productivity. An innovative way to address this challenge, which is currently lacking in most AR4D efforts, is critical. Under the existing circumstances, where due consideration for intra-plot heterogeneity is negligible, agronomists, farm managers and policy makers need to handle the yield values estimated using these methods and the management recommendations, thereof, with caution in decision-making.

## Declaration of Competing Interest

The authors report no declarations of interest.
