# Supplementary material for: Implications of intra-plot heterogeneity for yield estimation accuracy: Evidence from smallholder maize systems in Ethiopia
Source: Field Crops Res. 2021 Jun 15;267:108147. doi: 10.1016/j.fcr.2021.108147 (PMC8146729; doi:10.1016/j.fcr.2021.108147)
Supplement: Supplementary file 1 [file mmc1.docx]

# Supplemental materials

Table S 1: Summary of the backward Elimination algorithm using AIC model selection procedure. AIC = Akaike information criterion, RSS = Root sum of squares, SS = sum of squares,

| Variable |  | AIC |  | RSS |  | SS |  | R^2^ |  | Adj. R^2^ |
| --- | --- | --- | --- | --- | --- | --- | --- | --- | --- | --- |
| Full Model |  | 1541 |  | 7690 |  | 11712 |  | 0.6037 |  | 0.3106 |
| Planting date |  | 1537 |  | 7739 |  | 11663 |  | 0.6011 |  | 0.3119 |
| Planting date x NPS at planting |  | 1537 |  | 7746 |  | 11657 |  | 0.6008 |  | 0.3227 |
| Planting date x Urea split application |  | 1531 |  | 7765 |  | 11638 |  | 0.5998 |  | 0.3319 |
| Pesticides use x Planting date |  | 1527 |  | 7765 |  | 11638 |  | 0.5998 |  | 0.3319 |
| Legume in last 3 years |  | 1525 |  | 7765 |  | 11638 |  | 0.5998 |  | 0.3319 |
| Inter-plant spacing |  | 1525 |  | 7765 |  | 11638 |  | 0.5998 |  | 0.3319 |
| Distance to home |  | 1525 |  | 7765 |  | 11638 |  | 0.5998 |  | 0.3373 |
| Irrigation x NPS at planting |  | 1519 |  | 7765 |  | 11638 |  | 0.5998 |  | 0.3425 |
| Legume in last 3 years x Urea at planting |  | 1517 |  | 7765 |  | 11637 |  | 0.5998 |  | 0.3477 |
| NPS at planting x Herbicide use |  | 1517 |  | 7767 |  | 11636 |  | 0.5997 |  | 0.3527 |
| Urea at planting x NPS at planting |  | 1517 |  | 7768 |  | 11635 |  | 0.5996 |  | 0.3576 |
| Inter-plant spacing x Urea split application |  | 1511 |  | 7771 |  | 11632 |  | 0.5995 |  | 0.3623 |
| Inter-row spacing x NPS at planting |  | 1509 |  | 7773 |  | 11630 |  | 0.5994 |  | 0.3670 |
| Pesticides use x Irrigation |  | 1507 |  | 7776 |  | 11627 |  | 0.5992 |  | 0.3715 |
| Legume in last 3 years x Pesticide use |  | 1505 |  | 7781 |  | 11622 |  | 0.5990 |  | 0.3758 |
| Legume in last 3 years x Inter-row spacing |  | 1503 |  | 7794 |  | 11609 |  | 0.5983 |  | 0.3795 |
| Distance to home x Urea rate |  | 1501 |  | 7810 |  | 11593 |  | 0.5975 |  | 0.3828 |
| Legume in last 3 years x Urea split application |  | 1499 |  | 7827 |  | 11575 |  | 0.5966 |  | 0.3860 |
| Seed rate x Inter-plant spacing |  | 1497 |  | 7847 |  | 11555 |  | 0.5956 |  | 0.3889 |
| Distance to home x Urea split application |  | 1496 |  | 7878 |  | 11524 |  | 0.5940 |  | 0.3909 |
| Seed rate x Legume in last 3 years |  | 1494 |  | 7903 |  | 11500 |  | 0.5927 |  | 0.3934 |
| Legume in last 3 years x Distance to home |  | 1492 |  | 7932 |  | 11471 |  | 0.5912 |  | 0.3955 |
| Pesticides use x Urea at planting |  | 1491 |  | 7974 |  | 11428 |  | 0.5890 |  | 0.3966 |
| Inter-plant spacing x Distance to main road |  | 1489 |  | 8125 |  | 11278 |  | 0.5813 |  | 0.3939 |
| Legume in last 3 years x Inter-row spacing |  | 1488 |  | 8170 |  | 11233 |  | 0.5789 |  | 0.3947 |
| Irrigation x Herbicide use |  | 1487 |  | 8215 |  | 11187 |  | 0.5766 |  | 0.3956 |
| Inter-row spacing x Urea split application |  | 1486 |  | 8326 |  | 11077 |  | 0.5709 |  | 0.3958 |
| Inter-plant spacing x Urea rate |  | 1485 |  | 8411 |  | 10992 |  | 0.5665 |  | 0.3978 |
| Planting date x Urea at planting |  | 1485 |  | 8441 |  | 10962 |  | 0.5650 |  | 0.3996 |
| Irrigation x NPS rate |  | 1484 |  | 8507 |  | 10895 |  | 0.5615 |  | 0.3989 |
| Pesticide use x Urea split application |  | 1483 |  | 8558 |  | 10845 |  | 0.5589 |  | 0.3994 |
| Planting date x Urea rate |  | 1482 |  | 8605 |  | 10797 |  | 0.5565 |  | 0.3999 |
| Planting date x NPS rate |  | 1480 |  | 8667 |  | 10736 |  | 0.5533 |  | 0.3996 |

Table S 2: Levels of between fields and within fields variability captured by different yield estimation (sampling) methods. CV = Coefficient of variation (%).

|  |  | Between farm variability (% CV) | | | | |  | Intra-plot variability (% CV) | | | | |
| --- | --- | --- | --- | --- | --- | --- | --- | --- | --- | --- | --- | --- |
| Methods |  | Plant population |  | Cob Weight |  | Yield |  | Plant population |  | Cob Weight |  | Yield |
| M1 |  | 35.9 |  | 25.1 |  | 47.7 |  | 22.8 (8.4-86) |  | 22.5 (0 - 91) |  | 35.3 (10.4 - 99) |
| M2 |  | 36.6 |  | 28.2 |  | 51.0 |  | 19.0 (0 - 66) |  | 25.3 (0 - 87) |  | 25.0 (3.1 - 84) |
| M4 |  | 32.5 |  | 24.1 |  | 40.3 |  | 12.1 (0.5 - 62) |  | 17.4 (1.1 - 94) |  | 19.8 (0.9 - 69) |
| M5 |  | 49.3 |  | 22.0 |  | 39.0 |  | 20.4 (2.5 - 114) |  | 26.7 (9.5 - 73) |  | 24.43 (8-61) |

Table S 3: Summary of Average Marginal Effects (AME) of important variables whose main and interaction effects showed significant impact on intra-plot heterogeneity. SE = Standard Error, LCI = lower confidence interval, and UCI = upper confidence interval.

| Factors |  | AME |  | SE |  | Z-values |  | LCI |  | UCI |  |
| --- | --- | --- | --- | --- | --- | --- | --- | --- | --- | --- | --- |
| Late planting |  | 3.7 |  | 2.4 |  | 1.5 |  | -1.0 |  | 8.4 |  |
| Medium planting |  | 3.1 |  | 2.4 |  | 1.3 |  | -1.7 |  | 7.9 |  |
| Permanent conservation (Yes) |  | -0.4 |  | 1.2 |  | -0.4 |  | -2.9 |  | 2.0 |  |
| Inter-row spacing |  | 0.2 |  | 0.2 |  | 1.2 |  | -0.2 |  | 0.6 |  |
| Inter-plant spacing |  | 0.2 |  | 0.3 |  | 0.8 |  | -0.3 |  | 0.8 |  |
| Observable damage |  | 3.2 |  | 1.9 |  | 1.7 |  | -0.6 |  | 7.0 |  |
| Pesticide use |  | 4.5 |  | 3.3 |  | 1.4 |  | -1.9 |  | 11.0 |  |
| Herbicide use (Yes) |  | -3.0 |  | 1.6 |  | -1.9 |  | -6.1 |  | 0.1 |  |
| Distance to home |  | 0.0 |  | 0.0 |  | -0.4 |  | -0.1 |  | 0.1 |  |
| Distance to market |  | 0.1 |  | 0.0 |  | 2.5 |  | 0.0 |  | 0.1 |  |
| Distance to main road |  | -0.1 |  | 0.0 |  | -2.1 |  | -0.2 |  | 0.0 |  |
| Type of variety (Local) |  | 1.1 |  | 3.1 |  | 0.4 |  | -5.0 |  | 7.3 |  |
| Seed rate |  | 0.4 |  | 0.2 |  | 1.6 |  | -0.1 |  | 0.8 |  |
| Urea rate |  | 0.0 |  | 0.0 |  | -0.3 |  | 0.0 |  | 0.0 |  |
| Urea at planting (Yes) |  | 5.1 |  | 1.9 |  | 2.8 |  | 1.5 |  | 8.8 |  |
| Urea split application (Yes) |  | 3.4 |  | 2.4 |  | 1.4 |  | -1.4 |  | 8.2 |  |
| NPS rate |  | 0.0 |  | 0.0 |  | -1.3 |  | -0.1 |  | 0.0 |  |
| Spacing factor |  | -35.5 |  | 43.9 |  | -0.8 |  | -121.5 |  | 50.5 |  |
